# Human adipose-derived stem cells support the growth of limbal stem/progenitor cells

**DOI:** 10.1371/journal.pone.0186238

**Published:** 2017-10-11

**Authors:** Hua Mei, Sheyla González, Martin N. Nakatsu, Elfren R. Baclagon, Felix V. Chen, Sophie X. Deng

**Affiliations:** 1 Cornea Division, Stein Eye Institute, University of California, Los Angeles, California, United States of America; 2 UCLA College of Letters and Science, University of California, Los Angeles, California, United States of America; Instituto Butantan, BRAZIL

## Abstract

The most efficient method to expand limbal stem cells (LSCs) *in vitro* for clinical transplantation is to culture single LSCs directly on growth-arrested mouse fibroblast 3T3 cells. To reduce possible xenobiotic contamination from 3T3s, primary human adipose-derived stem cells (ASCs) were examined as feeder cells to support the expansion of LSCs *in vitro*. To optimize the ASC-supported culture, freshly isolated limbal epithelial cells in the form of single cells (SC-ASC) or cell clusters (CC-ASC) were cultured using three different methods: LSCs seeded directly on feeder cells, a 3-dimensional (3D) culture system and a 3D culture system with fibrin (fibrin 3D). The expanded LSCs were examined at the end of a 2-week culture. The standard 3T3 culture served as control. Expansion of SC-ASC showed limited proliferation and exhibited differentiated morphology. CC-ASC generated epithelial cells with undifferentiated morphology in all culture methods, among which CC-ASC in 3D culture supported the highest cell doubling (cells doubled 9.0 times compared to cells doubled 4.9 times in control) while maintained the percentage of putative limbal stem/progenitor cells compared to the control. There were few cell-cell contacts between cultured LSCs and ASCs in 3D CC-ASC. In conclusion, ASCs support the growth of LSCs in the form of cell clusters but not in single cells. 3D CC-ASC could serve as a substitute for the standard 3T3 culture to expand LSCs.

## Introduction

The integrity of human corneal epithelial cells is maintained by limbal stem cells (LSCs) [1–5], which are located at the basal limbal epithelium and surrounded by niche cells including limbal mesenchymal cells [6–8], melanocytes [9], and N-cadherin-expressing cells [10]. Loss of LSCs or their dysfunction may lead to limbal stem cell deficiency (LSCD) which present with corneal opacity, vascularization and conjunctivalization. Transplantation of *ex vivo* expanded LSCs to the LSCD eye has been reported as a successful therapy to treat LSCD [5, 11, 12]. A comprehensive review showed that the overall success rate is 76% from 583 patients [13].

The standard method to culture LSCs on 3T3 feeder cells that have been used in clinical study is cultivating single LSC directly on top of the growth-arrested 3T3 feeder cells [14]. Once sufficient amount of LSCs is achieved, the cultivated LSCs are transplanted onto the patient’s cornea after removing the abnormal epithelium and pannus. Although 3T3 fibroblast cells are growth-arrested and theoretically are not populated in patients, there are concerns about the mouse origin of the 3T3 feeder cells in clinical applications including contamination from xenogenic molecules, immuno-rejection, and potential interspecies viral transmission. It has been reported that human embryonic stem cells co-cultured with animal-derived serum and feeder cells express immunogenic nonhuman sialic acid [15]. Retinal pigment epithelial cells and iris pigment epithelial cells co-cultured on mitomycin C-treated 3T3 fibroblasts were found to express mouse collagen type I [16]. 3T3 cells have an endogenous retrovirus containing a 3600-bp region of xenotropic murine leukemia virus-related virus (XMRV) which are associated with human prostate cancer and chronic fatigue syndrome [17].

To replace the mouse fibroblast feeder cells, human amniotic membrane and human-derived feeder cells have been examined for their potential to support the growth of LSCs *in vitro*. Both intact and denuded amniotic membrane have been shown to support the growth of LSCs either in the form of tissue explants or cell suspension [13, 18–24], although donor variation exists [25, 26]. Human amniotic epithelial cells might support the growth of LSCs that contained uniformly p63-positive epithelial progenitor cells [27]. Human limbal mesenchymal cells and limbal melanocytes [28, 29], and bone marrow-derived mesenchymal stem cells (BM-MSCs) [9], have been reported to serve as feeder cells to culture LSCs *in vitro*.

Human adipose-derived stem cells (ASCs) are an easily accessible autologous stem cell source, have a higher frequency of mesenchymal stem cells than BM-MSCs [30], and have been shown to support the growth of many types of stem cells including human embryonic stem cells [31, 32], induced pluripotent stem cells [31], and LSCs [33]. ASCs could support the *in vitro* expansion of LSCs with a lower clonogenic capacity than 3T3 and the expanded LSCs express some putative limbal stem/progenitor cell markers [33]. However, the comparison between the ASC and 3T3 is limited to the colony-forming efficiency (CFE) and there is limited comparison on the stem cell phenotypes of cultured LSCs, which is crucial for pre-clinical development. In addition, only direct co-culture method was used and ASCs do not show superior capacity in supporting the growth of LSCs than 3T3 [33]. We previously reported that a 3 dimensional (3D) culture system, in which the LSCs and the 3T3 feeder cells were cultured on the opposite sides of a porous membrane, supported the growth of LSCs and significantly increased the cell proliferation of LSC cultured in the form of cell clusters [34]. Whether the 3D culture system can facilitate the ASC-supported culture was examined in this study. Fibrin gel, which has been used as a carrier for epithelial cell propagation *in vitro* and human transplantation [14, 35], was coated on the porous membrane. The cultured LSCs on fibrin could be directly transplanted into patients' eyes without extra retrieving steps from culture surface. In this study, the potency that ASCs support the growth of LSCs was compared to the standard culture on 3T3 cells, including cell doubling, expressions of putative stem cell markers including ATP-binding cassette sub-family G member 2 (ABCG2) [36], N-terminally truncated transcripts of p63 (∆Np63) [14, 37], N-cadherin [10] and cytokeratin (K) 14 [38], maturation marker K12 [39], and proliferation marker Ki67 [40]. Different forms of seeded LSCs and different culture methods were examined using ASC feeder cells to investigate which approach was the most optimal. The culture method using 3T3s that has been successfully used in clinical study, which is single LSCs cultured directly on 3T3 feeder cells, served as the control in all experiments.

## Materials and methods

### Human sclerocorneal tissue

Human sclerocorneal tissue was from the Lions Eye Institute for Transplant and Research (Tampa, FL) and the Illinois Eye Bank (Watson Gailey, Bloomington, IL). Tissue donors were aged from 20 to 65 years old. Experimentation on human tissue adhered to the tenets of the Declaration of Helsinki. The experimental protocol was evaluated and exempted by the University of California, Los Angeles Institutional Review Boards. The donors from whom the tissues were used in this study provided informed consent to being included of the study.

The tissues were preserved in Optisol (Chiron Ophthalmics, Inc., Irvine, CA), and the death-to-preservation time was less than 8 hours.

### Isolation, culture, and characterization of the primary ASCs

ASCs at passage 1 and 2 were a generous gift from Prof. Bruno Peault (Professor of Orthopedic Surgery, University of California, Los Angeles). The protocol of ASC isolation was described previously [41]. In brief, the lipoaspirate were incubated in RPMI 1640 (Cellgro, Corning, NY) containing 3.5% bovine serum albumin (Sigma-Aldrich, St. Louis, MO) and 1 mg/ml collagenase type II (Sigma-Aldrich) for 30 min at 37°C. After centrifugation, the adipocytes were discarded and the pellet was further incubated in red blood cell lysis buffer (eBioscience, San Diego, CA) to remove erythrocytes. The remaining cells were cultured in MEM-α (Gibco, Grand Island, NY) supplemented with 10% FBS (Invitrogen, Carlsbad, CA) and penicillin/streptomycin (Invitrogen), which are primary ASCs at passage 0. ASCs were subcultured at 1:6 ratio at around 80% confluence. ASCs at passage 4–6 were used for experiments.

Primary ASCs were characterized by the positive expression of MSC markers CD90 [42–44] and CD105 [42–44], negative expression of endothelial and hematopoietic stem/progenitor cell markers CD31 [45, 46] and CD34 [42–44] and negative expression of differentiation markers adiponectin [44, 47] (a marker of adipogenesis) and osteocalcin [44, 48] (a marker of osteogenesis).

### Isolation of limbal epithelial cells

LECs were isolated from corneoscleral rims as previously described [49]. In brief, the iris, endothelium, residual blood vessels, Tenon’s capsules, and conjunctiva were removed mechanically, followed by the digestion in 2.4 U/ml Dispase II (Roche, Indianapolis, IN) in SHEM5 growth medium (DMEM/F12 medium) (Gibco) supplemented with N-2 (Gibco), 2 ng/ml epidermal growth factor (EGF; Gibco), 8.4 ng/ml cholera toxin (Sigma-Aldrich), 0.5 μg/ml hydrocortisone (Sigma-Aldrich), 0.5% dimethyl sulfoxide (DMSO; Sigma-Aldrich), 5% fetal bovine serum (FBS, Invitrogen), penicillin/streptomycin (Invitrogen) and gentamicin/amphotericin B (Invitrogen) for 2 hours at 37°C. Epithelial cell sheets were mechanically scraped from the limbus and pipetted for several times to break the cell sheets into smaller cell clusters, which were usually a mixture of mainly single cells and some small cell clusters (around 2 to 20 cells/cluster, as shown in S1 Fig). Some cell clusters were further digested with 0.25% trypsin and 1 mM EDTA (Gibco) for 10 min at 37°C to obtain single-cell suspension. LECs, either in the form of cell clusters (composed of both single cells and small cell clusters) or single-cell suspension, were seeded at a density of 300 cells/cm^2^.

### Cell culture of LSCs

The direct and 3D culture methods were following the protocol as previously described [34]. In brief, in the direct culture method, the LECs were seeded and cultured directly on the feeder cells. In the 3D culture method, the LECs were cultured on the top side of a 1μm pore polyethylene terephthalate (PET) membrane and the feeder cells were cultured on the bottom side of the membrane [34]. In the fibrin 3D culture method, a 1 to 2mm-thick layer of fibrin gel (Baxter, Deerfield, IL) was coated on top of the porous membrane; the feeder cells were seeded at the bottom side of the membrane and the LECs were seeded on top of the fibrin gel. Subconfluent murine 3T3 cells (from Howard Green, Harvard Medical School, Boston, MA, USA) were growth-arrested with 4 μg/ml of mitomycin C (Sigma-Aldrich) for 2 h, and plated at 3 x 10^4^ cells/cm^2^ as feeder cells. Subconfluent ASCs were treated with 4 μg/ml of mitomycin C (Sigma-Aldrich) for 2 h, and plated at 5 x 10^3^ cells/cm^2^ (an optimized density to support epithelial growth) as feeder cells. The LECs from the same donor were used for culture in different conditions for each experiment to minimize donor variation. The cells were cultured in SHEM5 growth medium for 14 days before harvest. The growth medium was refreshed every 2–3 days. The cell doubling was calculated as log_2_(number of epithelial cells harvested at day 14/ number of cells seeded).

### Collection of expanded LSCs at the end of 14-day culture

For 3T3 SC control, SC-ASC, and CC-ASC culture, in which LSCs and feeder cells were grown on the same side of plates/plate inserts, the LSCs and the feeder cells were incubated in Versene (Gibco) for 3 min; then the feeder cells were washed away by pipetting and the epithelial colonies remained attached to the plates/plate inserts. For 3D SC-ASC, 3D CC-ASC, and fibrin 3D CC-ASC culture, in which LSCs and feeder cells were grown on the opposite sides of plate inserts, the feeder cells were removed by mechanical scraping with a cell scraper (Fisher Scientific) and the epithelial colonies remained attached on the top side of plate inserts. The remaining epithelial cells were incubated in 0.25% trypsin and 1 mM EDTA (Gibco) for 5 to 8 min at 37°C and collected for further analysis.

### Success rate of culture

The success rate of culture, which was to examine whether the culture supported a consistent growth of LSCs from different donors, was presented as the number of donors showing LSC growth out of the total number of donors examined.

### RNA isolation, reverse transcription and quantitative real-time PCR

RNA was extracted from harvested LECs (RNeasy Mini Kit, Qiagen, Valencia, CA), treated with DNase (DNA-free kit, Ambion, Austin, TX), and reverse-transcribed into cDNA (SuperScript II, Invitrogen) according to the manufacturers’ protocols. mRNA transcripts were quantified by using the Kapa Sybr Fast qPCR kit (Kapa Biosystems, Woburn, MA). Cycle conditions were as follows: the reactant was denatured for 20 s at 95°C; amplified for 40 cycles (temperatures in each cycle were 95°C for 3 s, 60°C for 20 s, and 72°C for 8 s); and subjected to a melting curve program to obtain the dissociation curves. The primers used in quantitative real-time PCR (qRT-PCR) were listed in S1 Table.

### Immunocytochemistry and quantitation

Cells were cytospined onto slides by a cytocentrifuge (Cytofuge; Fisher Scientific, Hampton, NH) and stored at -80°C until use. Cytospin slides were fixed with 4% paraformaldehyde at room temperature for 10 min and washed 3 times with phosphate-buffered saline (PBS). Samples were blocked and permeabilized in PBS containing 1% BSA and 0.5% Triton X-100 (Sigma-Aldrich) for 30 min at room temperature and incubated with the primary antibody diluted in PBS containing 1% bovine serum albumin (BSA) and 0.1% Triton X-100 overnight at 4°C in a moist chamber. Slides were washed 3 times with PBS, incubated with the secondary antibody diluted in PBS containing 1% BSA and 0.1% Triton X-100 at room temperature for 1 h, and washed with PBS for three times. Nuclei were labeled with Hoechst 33342 (4 μg/ml; Invitrogen) at room temperature for 15 min, washed 5 times with PBS, and mounted in Fluoromount medium (Sigma). The primary antibodies and their dilution ratios are listed in S2 Table.

Images were taken by a confocal microscope (Confocal Laser Scanning Microscopy; Olympus, San Jose, CA) and an image capture system (Fluoview FV10-ASW 3.1 Viewer; Olympus). The nuclear intensity of p63α was analyzed by the Definiens Tissue Studio software (Larchmont, NY).

### Analysis of cell-cell contact in the 3D CC-ASC culture by high resolution light microscopy and electron microscopy

At the end of the 3D CC-ASC culture, membranes with cells were carefully removed, fixed with 2% glutaraldehyde (Electron microscope Sciences, Hatfield, PA) and 2% paraformaldehyde (Electron microscope Sciences) in 0.1 M cacodylate buffer, washed with 0.1 M cacodylate buffer, osmicated for 1 h, washed extensively and embedded in Epon resin (Momentive Specialty Chemicals, Houston, TX). Tissue was sectioned at 1 μm and stained with toluidine blue (Sigma) for light microscopy. The number of the pores showing no visible cell extension, showing cell extension from epithelial cells (non-contacting extension), showing cell extension from ASC feeder cells (non-contacting extension), or showing extension throughout pores connecting epithelial cells to ASC feeder cells (contacting extension) were manually counted. For electron microscopy, tissue was sectioned at 60 nm and stained in 8% uranyl acetate (Ted Pella Inc., Redding, CA) for 15 min followed by a solution of 0.4% lead citrate (Ted Pella Inc.) and 0.4% sodium hydroxide (Fisher Scientific) for 2 min. The images were taken by a JEOL JEM1200-EX transmission electron microscope (JEOL, Peabody, MA).

### Statistical analysis

Student's *t*-test was performed to analyze the data. Error bar represent the standard error of the mean (SEM) from 3–5 experiments. P values ≤ 0.05 were considered statistical significant.

## Results

### Characterization of the ASCs

**Fig 1:**
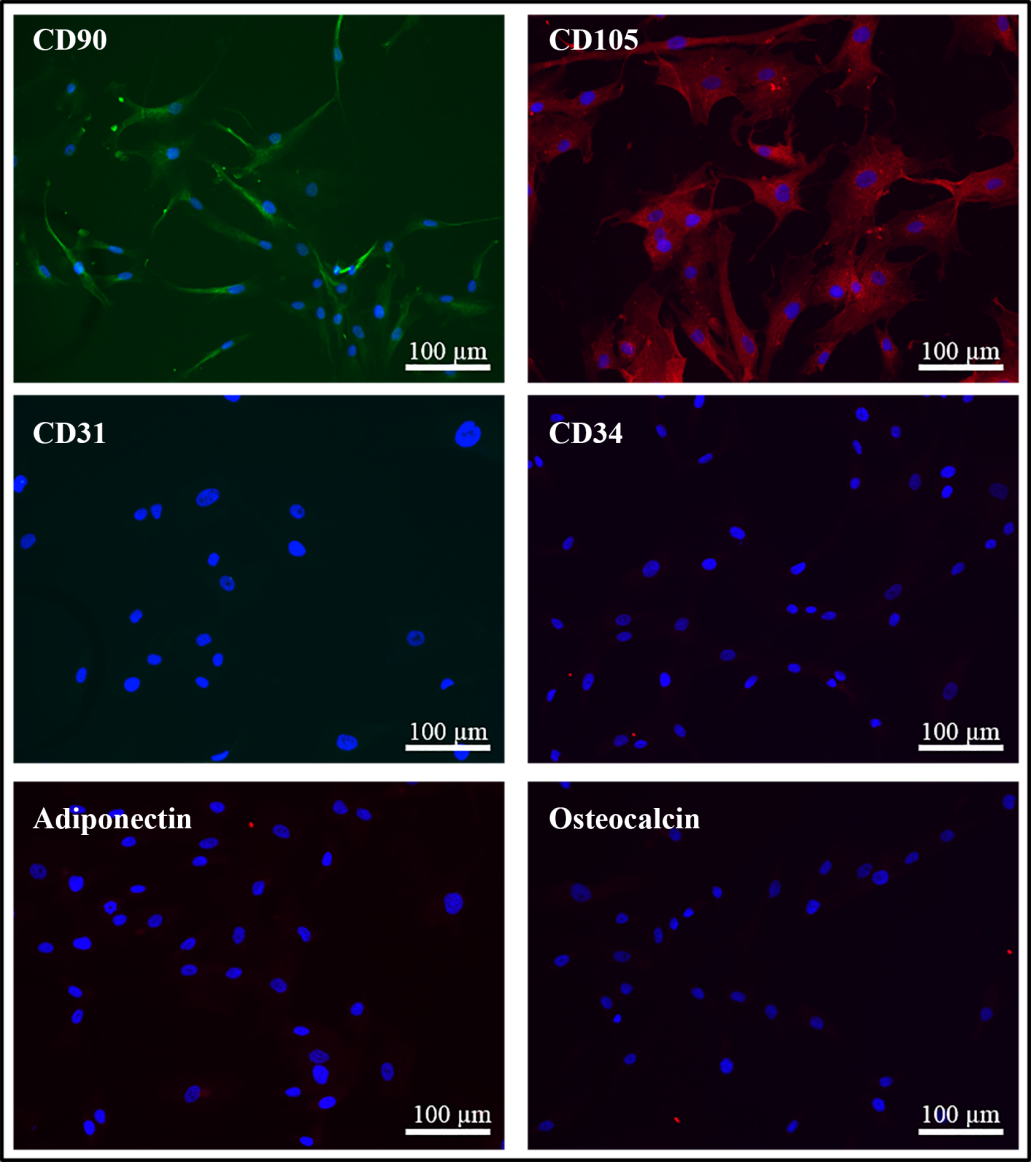
Characterization of primary ASCs. The expression of CD90, CD105, CD31, CD34, adiponectin, and osteocalcin was examined by immunocytochemistry in the primary ASCs at passage 4–6. Scale bar = 100 μm.

Human primary ASCs expressed the mesenchymal stem cell (MSC) markers including Cluster of Differentiation (CD) 90 and CD105 (Fig 1). The cells showed negative expression of endothelial and hematopoietic stem/progenitor cell markers, CD31 and CD34, respectively (Fig 1). There was no detectable expression of adiponectin, a differentiation marker of adipogenesis or osteocalcin, a differentiation marker of osteogenesis (Fig 1).

### Optimization of the cell density of ASCs as feeder cells

**Fig 2:**
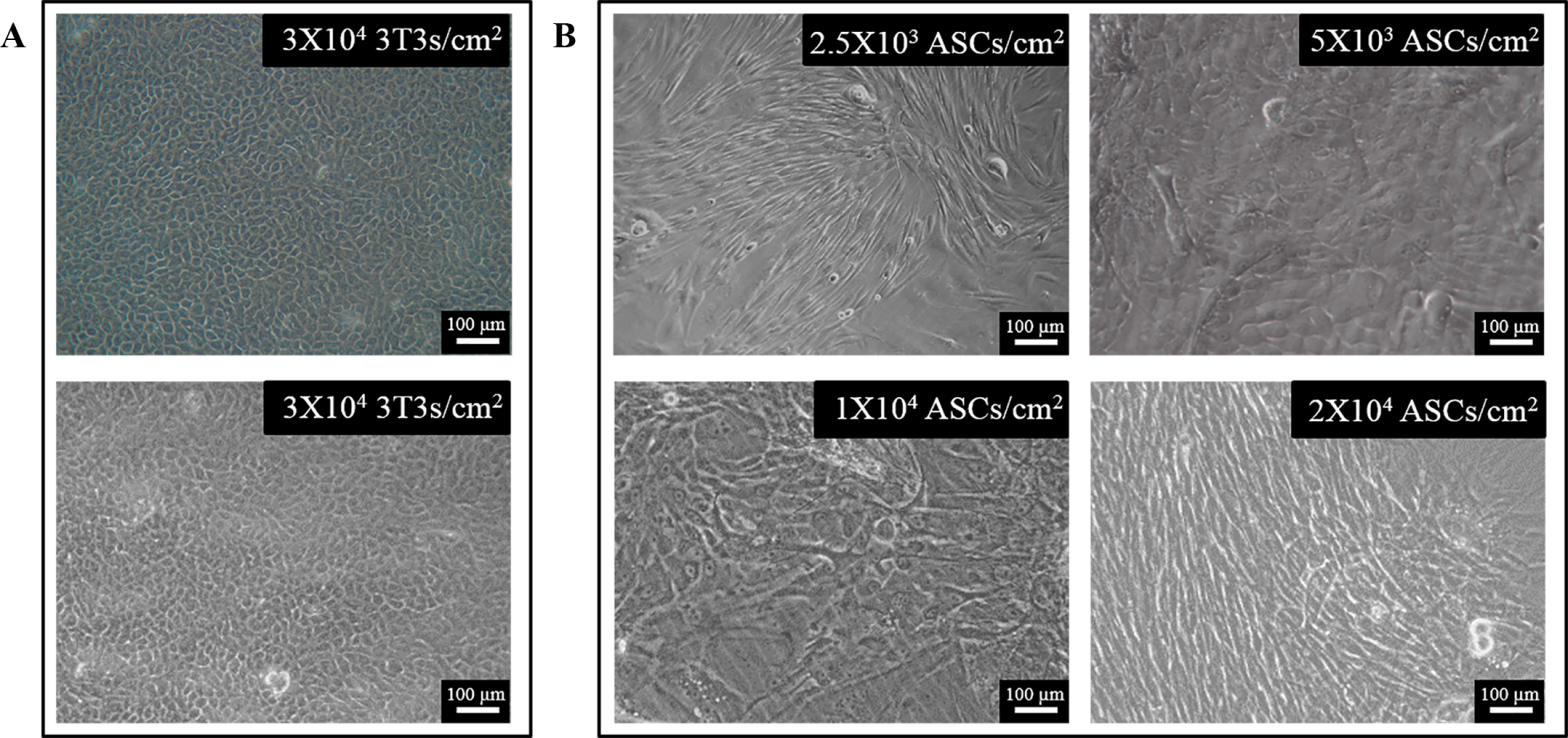
Optimization of the cell density of ASCs as feeder cells. (A) Representative morphology of LSCs cultured on 3 x 10^4^ 3T3 cells/cm^2^ for 2 weeks. (B) Representative morphology of LSCs cultured on gradient densities of ASCs for 2 weeks. The density of feeder cells was labeled at the top right corner of each image. Scale bar = 100 μm.

LSCs in single cell suspension were cultured directly on gradient densities of ASCs, i.e. 2.5 x 10^3^, 5 x 10^3^, 1 x 10^4^, and 2 x 10^4^ cells/cm^2^, for 2 weeks. The densities of 5 x 10^3^ and 1 x 10^4^ ASCs/cm^2^ supported epithelial growth (Fig 2B), although the cultured LSCs were not as cuboidal and compact as the LSCs cultured on 3T3 cells (Fig 2A). The lower density of 2.5 x 10^3^ ASCs/cm^2^ and the higher density of 2 x 10^4^ ASCs/cm^2^ failed to support epithelial expansion and favored the growth of fibroblast-like cells instead (Fig 2B). Between the two densities, 5 x 10^3^ and 1 x 10^4^ ASCs/cm^2^, the former density seemed to support slightly better epithelial growth and was chosen for the following experiments.

### ASCs do not support the growth of LSCs in single cell suspension

**Fig 3:**
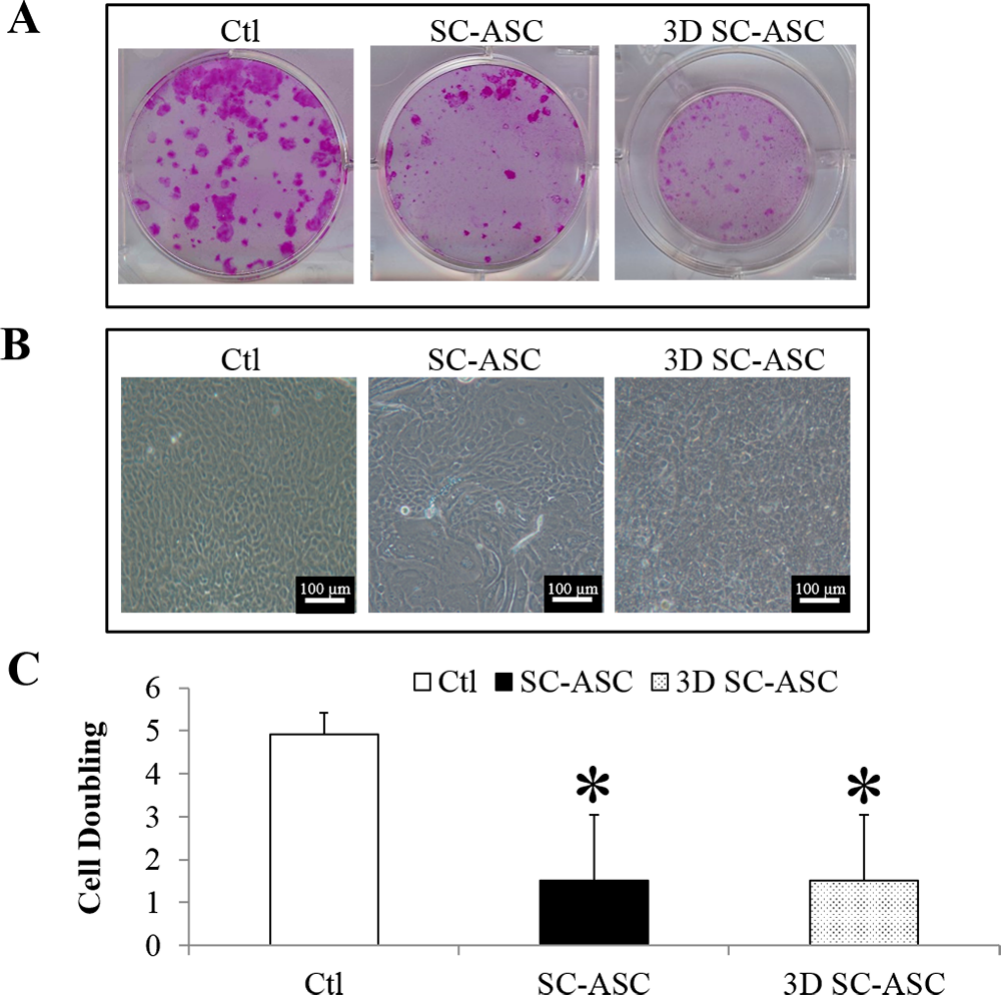
Single-cell suspension of limbal epithelial cells cultured on ASCs. (A) Cell morphology of cultured LSCs. The images for SC-ASC and 3D SC-ASC were from the 33% culture which supported epithelial expansion. (B) Cell morphology of cultured LSCs. The images for SC-ASC and 3D SC-ASC were from the 33% culture which supported epithelial expansion. (C) Cell doubling of limbal epithelial cells. *: p<0.05 in comparison with results of control. Ctl: control. SC-ASC: single cell suspension of LECs cultured directly on ASCs. 3D SC-ASC: single cell suspension of LECs cultured on ASCs using the 3D method. Scale bar = 100 μm.

Single cell suspension of LSCs were cultured on ASCs using the direct (SC-ASC) and 3D methods (3D SC-ASC) for 2 weeks. Single LECs cultured directly on 3T3 feeder cells (SC-3T3) served as the control. The LECs in the control group had a consistent growth (100%, 3 out of 3 donors) and a compact and cuboidal undifferentiated epithelial morphology (Fig 3B). SC-ASC and 3D SC-ASC cultures did not grow consistently that 67% of the culture (2 out of 3 donors) failed to grow and only 33% culture (1 out of 3 donors) showed epithelial growth. The expanded epithelial cells from the 33% culture formed small colonies (Fig 3A) and showed an uneven, flattened, and differentiated morphology (Fig 3B). Cell doubling in the SC-ASC and 3D SC-ASC cultures were significantly lower than the control (Fig 3C). There was no difference in cell doubling between the SC-ASC and 3D SC-ASC cultures. Due to the inconsistent growth and differentiated morphology, single LSCs cultured on ASCs were abandoned from further investigation.

### ASCs support the growth of LECs in cell clusters

**Fig 4:**
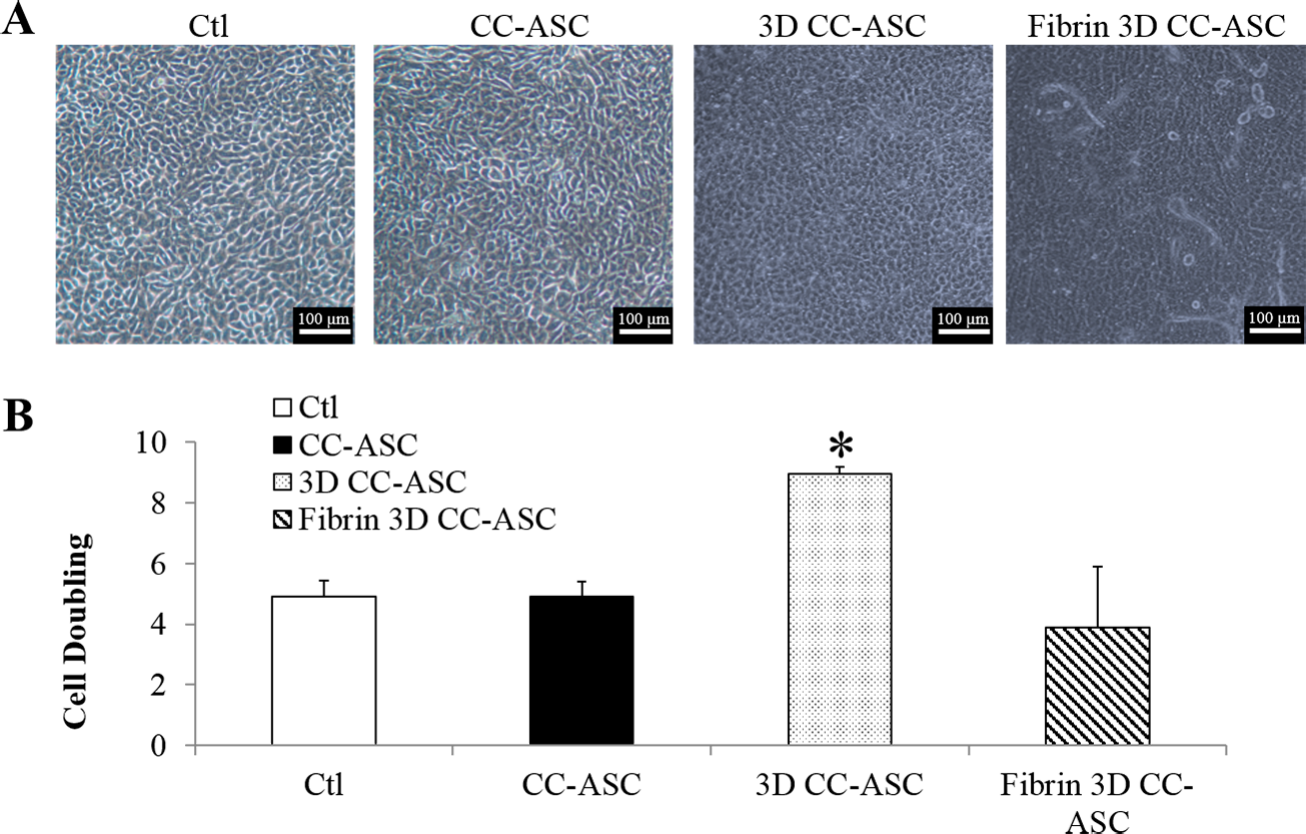
Cell clusters of limbal epithelial cells cultured on ASCs. (A) Cell morphology of cultured LSCs. The image for fibrin 3D CC-ASC was from the 33% culture which supported epithelial expansion. (B) Cell doubling of limbal epithelial cells. *: p<0.05 in comparison with results of control, CC-ASC, and fibrin 3D CC-ASC cultures. Ctl: control. CC-ASC: cell clusters of LECs cultured directly on ASCs. 3D CC-ASC: cell clusters of LECs cultured on ASCs using the 3D method. Fibrin 3D CC-ASC: cell clusters of LECs cultured on ASCs using the fibrin 3D method. Scale bar = 100 μm.

We have previously shown that LSCs grew better in the form of cell clusters than single cells in 3T3-supported culture [34]. Cell clusters of LSCs were then cultured on ASCs using the direct (CC-ASC), 3D (3D CC-ASC) and fibrin 3D (fibrin 3D CC-ASC) methods. CC-ASC and 3D CC-ASC supported a consistent (100%, 5 out of 5 donors) cell growth and produced a compact and cuboidal epithelial morphology, which was comparable to that in the control (SC-3T3 culture) (Fig 4A). However, the cells cultured in fibrin 3D CC-ASC did not always proliferate well. 33% culture (1 out of 3 donors) of fibrin 3D CC-ASC grew well and 67% culture (2 out of 3 donors) barely had proliferation. 3D CC-ASC had the consistently highest cell doubling, in which cell were doubled 9.0 times, compared to cells doubled 4.9 times in control (p<0.05), 4.9 times in CC-ASC (p<0.05), and 3.9 times in fibrin 3D CC-ASC (p<0.05) (Fig 4B). There was no difference on cell doubling among CC-ASC, fibrin 3D CC-ASC, and fibrin 3D CC-ASC (Fig 4B).

**Fig 5:**
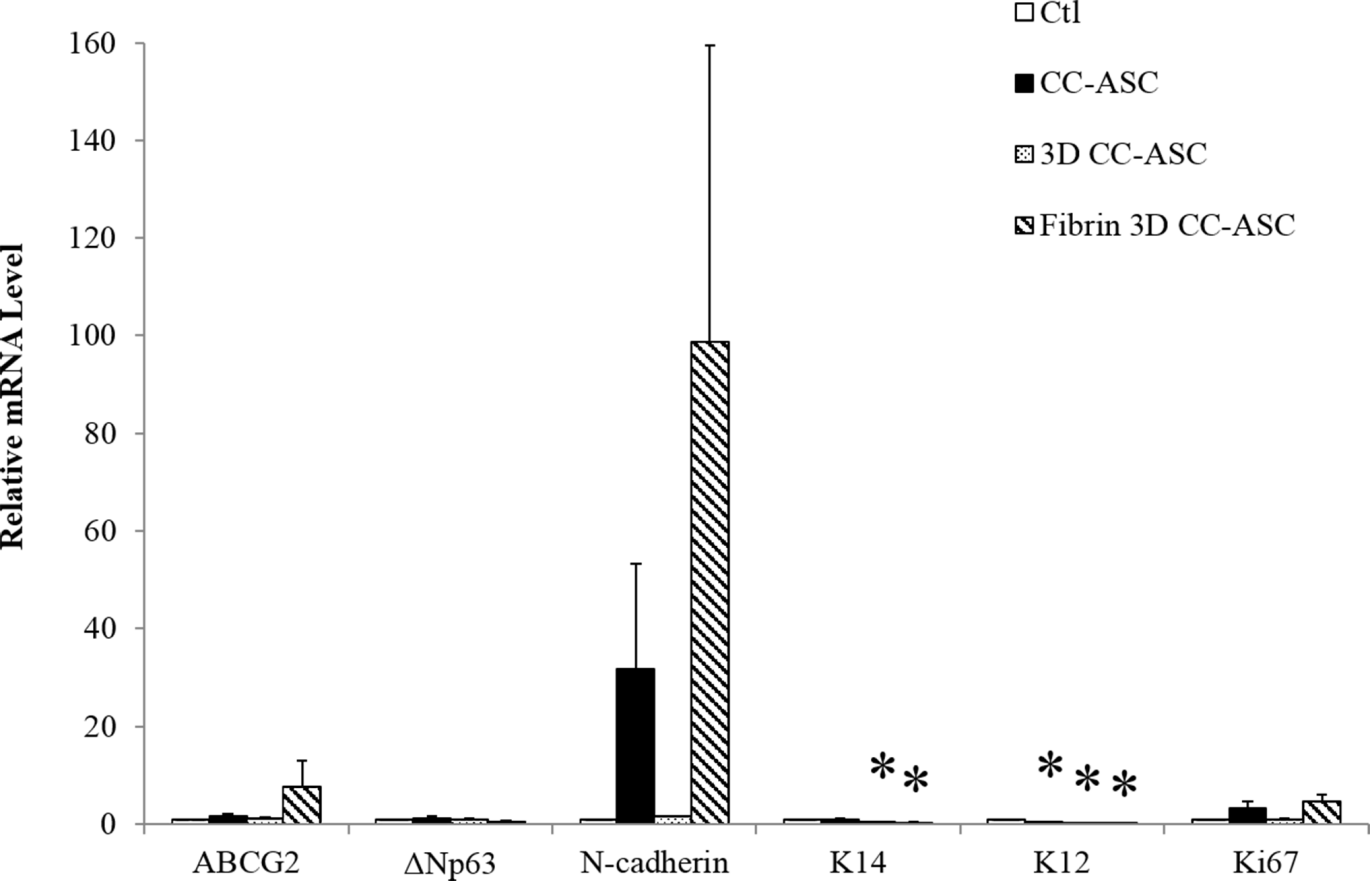
Relative mRNA levels of the putative stem cell markers and maturation marker in cultured LEC cell clusters with ASCs as evaluated by qRT-PCR. The expression of each marker was normalized to the expression of housekeeping gene GAPDH and the value of the control group was designated 1. *: p<0.05 in comparison with results of the control group. Ctl: control. CC-ASC: cell clusters of LECs cultured directly on ASCs. 3D CC-ASC: cell clusters of LECs cultured on ASCs using the 3D method. Fibrin 3D CC-ASC: cell clusters of LECs cultured on ASCs using the fibrin 3D method.

The stem cell phenotype of cultured LECs was characterized by qRT-PCR. Compared to the control (SC-3T3 culture), LECs cultured on ASCs expressed a similar mRNA level of ABCG2, ΔNp63 and N-cadherin (putative LSC markers) in all three culture methods, a significantly lower level of K14 (a putative LSC marker) in 3D and fibrin 3D methods (decreased by 54% and 72%, respectively, p<0.05) and a significantly lower level of K12 (a differentiation marker) in the direct, 3D and fibrin 3D methods (decreased by 65%, 85% and 90%, respectively, p<0.05) (Fig 5). There was no difference on the mRNA level of Ki67 (a proliferation marker) between the control and the three culture methods using ASCs as feeder cells (all p>0.05, Fig 5).

**Fig 6:**
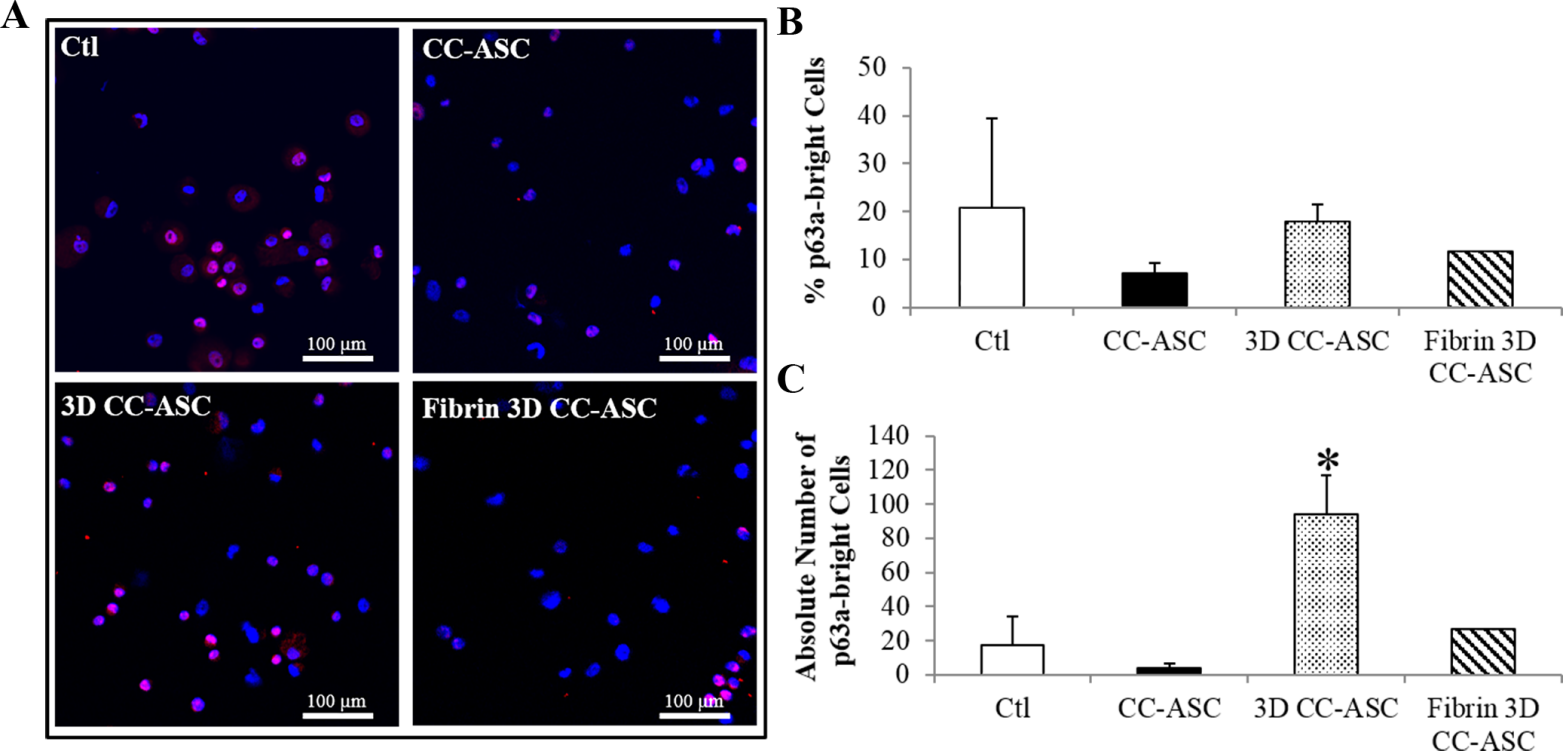
Expression of p63α in cultured LEC cell clusters with ASCs evaluated by immunocytochemistry. (A) Representative images showing the expression of p63α in cultured LECs. (B) The percentages of p63α-bright cells in cultured LECs. (C) The absolute numbers of p63α-bright cells in cultured LECs. The absolute number of p63α-bright cells = the percentage of p63α-bright cells x (number of cells harvested/number of cells seeded). *: p<0.05 in comparison with results of control. Ctl: control. CC-ASC: cell clusters of LECs cultured directly on ASCs. 3D CC-ASC: cell clusters of LECs cultured on ASCs using the 3D method. Fibrin 3D CC-ASC: cell clusters of LECs cultured on ASCs using the fibrin 3D method. Scale bar = 100 μm.

**Fig 7:**
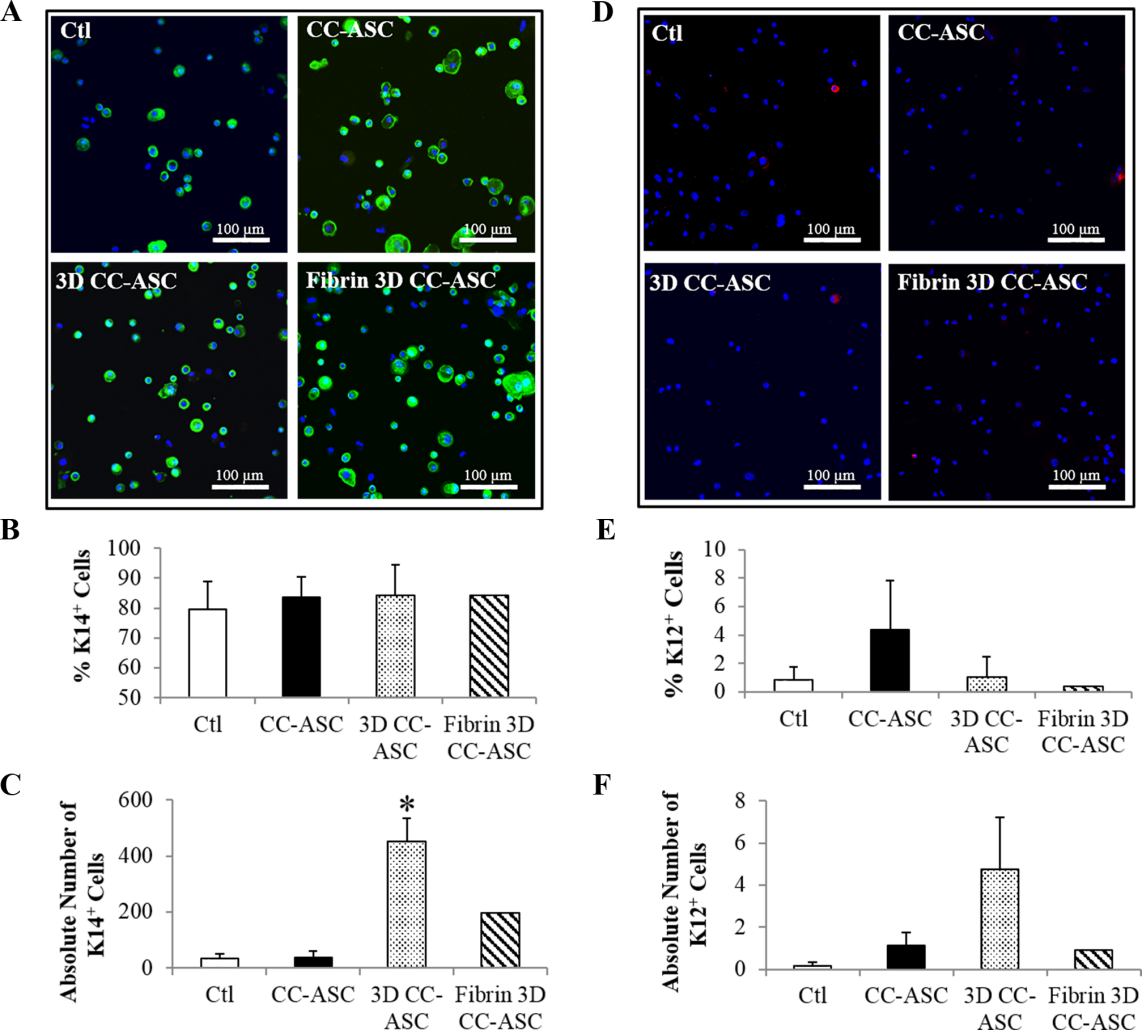
Expression of K14 and K12 in cultured LEC cell clusters with ASCs evaluated by immunocytochemistry. (A) Representative images showing the expression of K14 in cultured LECs. (B) The percentages of K14^+^ cells in cultured LECs. (C) The absolute numbers of K14^+^ cells in cultured LECs. (D) Representative images showing the expression of K12 in cultured LECs. (E) The percentages of K12^+^ cells in cultured LECs. (F) The absolute numbers of K12^+^ cells in cultured LECs. The absolute number of K14^+^ or K12^+^ cells = the percentage of K14^+^ or K12^+^ cells x (number of cells harvested/number of cells seeded). *: p<0.05 in comparison with results of control. Ctl: control. CC-ASC: cell clusters of LECs cultured directly on ASCs. 3D CC-ASC: cell clusters of LECs cultured on ASCs using the 3D method. Fibrin 3D CC-ASC: cell clusters of LECs cultured on ASCs using the fibrin 3D method. Scale bar = 100 μm.

The phenotype of cultured LSCs was further examined by the protein expression using immunocytochemistry. LSC cell clusters cultured on ASCs had comparable percentages of p63α-bright and K14^+^ progenitor cells in all three culture methods compared to the control (Figs 6B, 7B and 7E). Percentage of p63α-bright cells was shown to correlate with clinical success rate [14]. Therefore, we used percentage of p63α-bright cells to evaluate the quality of LSCs expanded. Because 3D CC-ASC had the highest cell doubling as shown in Fig 3B, it generated significantly higher absolute numbers of p63α-bright (4.4-folds higher, p<0.05) and K14^+^ cells (13-folds higher, p<0.05) than the control (Figs 6C and 7C). There was no significant difference in the absolute number of K12^+^ cells between the control and all ASCs cultures (Fig 7F).

### Few direct cell-cell contacts between the LSCs and the ASC feeder cells in 3D CC-ASC culture

**Fig 8:**
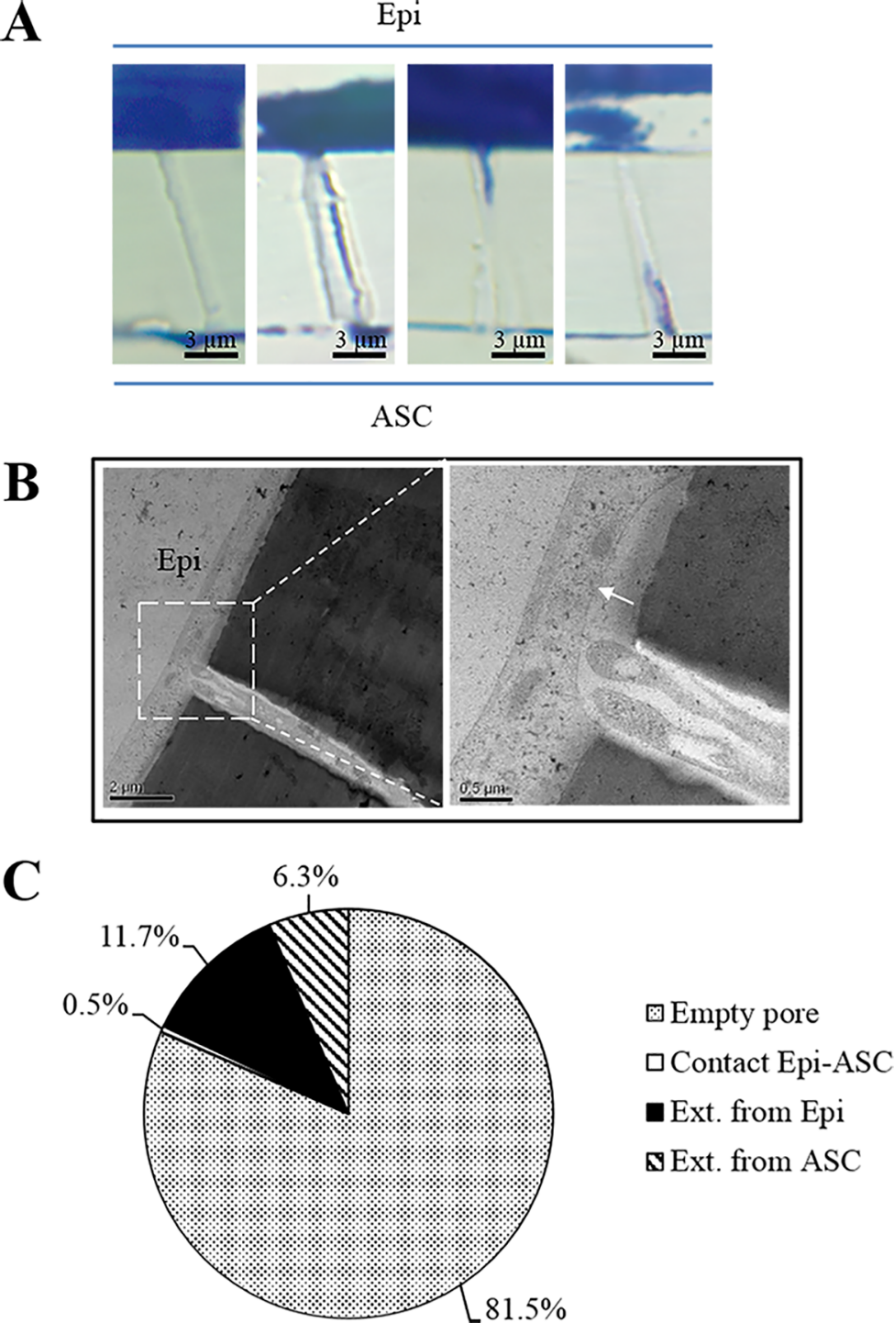
Few cell-cell contacts between the epithelial cells and the ASC feeder cells in 3D CC-ASC culture. (A) Representative images showing an empty pore, a pore with possible cell-cell contact, a pore with no cell-cell contact but with cell extension from the epithelial cells, and a pore with no cell-cell contact but with cell extension from the ASC feeder cells, respectively. (B) An image showing the cell-cell contact between LSCs and ASC feeder cells using electron microscopy. White arrow indicates the interface of cell-cell contact. (C) Percentages of pores with empty content, cell-cell contact, no contact but with cell extension from epithelial cells, and no contact but with cell extension from ASC feeder cells in the 3D CC-ASC culture. Epi: cultured epithelial cells. Scale bar = 3 μm.

To study whether there was any direct cell-cell contact between the LSCs and the ASC feeder cells, 3D CC-ASCs were fixed, sectioned across the pores in the PET membrane, stained and counted. Over 400 pores were examined and 0.5% of the pores showed direct cell-cell contact (Fig 8A, 8B and 8C). Majority (81.5%) of the pores showed no visible cell extension from either the epithelial or the ASC feeder cells. There were 18% pores containing cell extension either from the epithelial cells (11.7%) or from the ASC feeder cells (6.3%) (Fig 8C); however, the extensions failed to reach the cells on the other side of the membrane (Fig 8A).

## Discussion

ASCs can be easily harvested in large quantity from white adipose tissue through minimally invasive liposuction procedure [50, 51] and are well characterized [52–54]. ASCs could serve as feeder cells for many cell types including neuronal cells [55], melanocytes [56], and various types of epithelial stem cells including LSCs [33]. Our results showed that ASCs did not support the growth of LSCs in the form of single cell suspension as evident by the differentiated morphology and poor and inconsistent cell expansion. However, ASCs supported the growth of LSCs in the form of cell clusters. To access the quality of our culture, cell morphology, cell doubling, and expression of putative stem cell markers and maturation marker were examined. CFE was not quantitatively analyzed in this study because holoclones formed from limbal stem cells could not be distinguished from meroclones by colony size or shape [57]. Instead, the expression of p63α, which has been well characterized to predict holoclones [37] and shown to correlate with clinical success [14], was examined. LSC cell clusters cultured with ASCs were compact and small in size, and contained comparable percentages of p63α-bright, K14^+^, and K12^+^ cells in all three culture methods compared to the 3T3 culture (control) although there was a reduced expression of K14 and K12 in mRNA levels. The discrepancies between the data from immunocytochemistry and those from qRT-PCR on K12 and K14 expression indicate a poor correlation between the mRNA and protein expressions. The poor correlation is observed genome-widely in both bacteria and eukaryotes. Approximately 60% of the variation in protein concentration cannot correlate to the mRNA abundance [58]. The weak correlation between mRNA and protein levels reflects the precise control on gene expression at multiple levels, including transcript stability, post-translational modification, intracellular trafficking and packaging, and/or pathway-specific degradation. The transcriptional profile, translational profile, and even the discrepancies between them, may distinguish a type of cells from other populations and serve as cell signatures.

Compared to the human limbal mesenchymal cell-supported culture and human bone marrow stromal cell-supported culture which generate similar or 3-fold more LSCs respectively than the 3T3 culture at their optimized conditions [59, 60], 3D CC-ASC supported a remarkably high cell doubling of LSCs, in which cells were doubled 9 times compared to the cells doubled 4.9 times in control. In other words, 3D CC-ASC generated 14.3-fold more LSCs than 3T3 control. Meanwhile, 3D CC-ASC maintained a comparable percentage of LSC/progenitor cell population compared to 3T3 control, which led to significantly higher absolute numbers of p63α-bright and K14^+^ cells in cultured LSCs. The fact that 3D CC-ASC could support a significantly higher cell doubling of LSCs while maintaining the percentage of LSCs/progenitor cells is of great clinical significance, which enables the generation of sufficient amount of expanded LSCs for ransplantation from fewer cells derived from small biopsies. There were few cell-cell contacts between the LSCs and the ASCs in 3D CC-ASC culture thus limiting the contamination from feeder cells. These results suggest that expanding LSC cell clusters on ASCs using the 3D culture method may be an appropriate substitute for the 3T3 culture method.

The mRNA levels of Ki67, a proliferation marker, were comparable between the SC-3T3 control culture and the three culture methods in CC-ASC cultures in Fig 5, which was not consistent with the actual cell doubling in Fig 4B. One possible explanation is that the LSCs in the 3D CC-ASC culture may reach confluence at the end of 2-week culture and may stop the highly proliferative state at the time point of cell harvesting for mRNA quantitation.

The reason why 3T3 cells support the expansion of LSCs in single cell suspension whereas ASCs do not needs to be elucidated. One possible explanation is ASCs secret different molecules. It has been reported that ASCs showed a different mRNA expression pattern of secretary molecules that are known to regulate epithelial stem cells including pleiotrophin, cystatin C, hepatocyte growth factor, keratinocyte growth factor, Insulin-like growth factor 1α compared to 3T3 cells [33].

Another explanation may be the separation of LSCs from their neighboring cells and/or the cleavage of membrane proteins of LSCs by trypsin/EDTA that are important for the survival or attachment of LSCs. Previous reports showed that when single LECs which contained the LSC population and the LSC niche cells were seeded at a high density (5x10^4^ cells/cm^2^), the reunion of LSCs with their niche cells occurred and promoted the propagation of LSCs *in vitro* without 3T3 feeder cells [8]. However, when single LSCs were seeded at a lower density (300 cells/cm^2^) and single LSCs were separated sparsely from the niche cells, there was no cell growth without 3T3 feeder cells [61]. Similar to the niche cells, 3T3 cells support the single LSC growth under direct feeder-LSC contact, however they do not support the growth when the LSCs are cultured separately in the overhanging cell culture inserts [62]. These findings support the notion that LSCs need to be in direct contact or in close proximity with the niche cells or 3T3 feeder cells to survive and proliferate *in vitro*. In other words, niche cells and 3T3 feeder cells provide niche factors that signal through cell-cell contact or within a short distance to support the survival and proliferation of LSCs *in vitro*. This hypothesis that LSCs need to be in direct contact or in close proximity with the niche cells or feeder cells to survive and proliferate can also be used to explain the inferior performance of fibrin 3D CC-ASC culture. The 1–2 mm thick fibrin destructs the close proximity between ASC feeder cells and LSCs and may also block the cell-cell contact between the ASCs and LSCs, thus less niche factors from feeder cells could travel this distance through the gel and reach the LSCs to support their survival and proliferation. Interestingly, single LSCs were able to grow into transplantable cell sheets on denuded amniotic membrane without feeder cells [63], which suggests that amniotic membrane may contain the essential niche signals for the *in vitro* survival and proliferation of LSCs. In addition, trypsin, as a serine protease, cleaves outer membrane peptide chains mainly at the carboxyl side of lysine and arginine. The process of trypsinization to make single LSCs suspension may damage some membrane or membrane-associated molecules on LSCs which in turn impair the stem cell survival and adhesion.

In summary, human ASCs have the capacity to support the expansion of LSCs *in vitro*. A fine-tuned ASC-supported culture system including seeding LEC clusters and using the 3D culture method can achieve a significantly high cell expansion meanwhile maintaining the percentage of putative limbal stem/progenitor cell population with minimal cell-cell contacts from feeder cells, which enables the generation of sufficient amount of LSCs for transplantation from fewer cells derived from small biopsies while minimizing cell contamination from feeder cells. Therefore, 3D CC-ASC appears to be a good substitute for the standard 3T3 culture to expand LSCs *in vitro* for clinical application.

## Supporting information

S1 Fig Representative images showing the cell clusters of LECs. The cell clusters of LECs were obtained by Dispase digestion of corneoscleral rim followed by mechanical scraping and pippeting. The cell clusters of LECs were composed of mainly single cells and some small cell clusters (usually around 2 to 20 cells/cluster). The cell clusters in dashed rectangles are enlarged at the sides of the images. Scale bar = 100 μm.
(TIF)

S1 Table Primers used in qRT-PCR. (DOCX)

S2 Table Primary antibodies used in immunocytochemistry. (DOCX)
