# Supplementary figures and images for: Human adipose-derived stem cells support the growth of limbal stem/progenitor cells
Source: PLoS One. 2017 Oct 11;12(10):e0186238. doi: 10.1371/journal.pone.0186238 (PMC5636133; doi:10.1371/journal.pone.0186238)

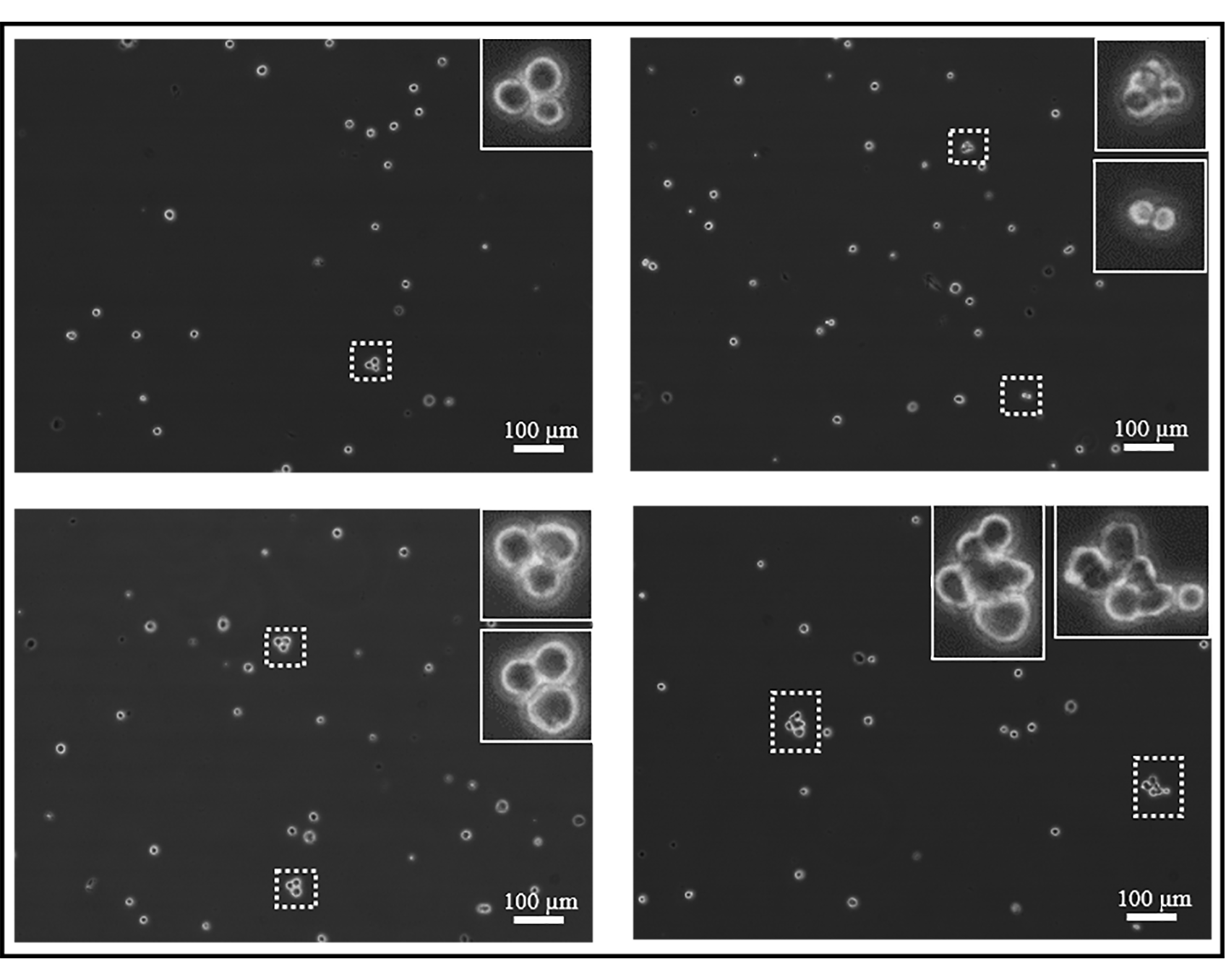

Supplement: S1 Fig — The cell clusters of LECs were obtained by Dispase digestion of corneoscleral rim followed by mechanical scraping and pippeting. The cell clusters of LECs were composed of mainly single cells and some small cell clusters (usually around 2 to 20 cells/cluster). The cell clusters in dashed rectangles are enlarged at the sides of the images. Scale bar = 100 μm. (TIF) [file pone.0186238.s001.tif]
