# Supplementary material for: Human adipose-derived stem cells support the growth of limbal stem/progenitor cells
Source: PLoS One. 2017 Oct 11;12(10):e0186238. doi: 10.1371/journal.pone.0186238 (PMC5636133; doi:10.1371/journal.pone.0186238)
Supplement: S1 Table — (DOCX) [file pone.0186238.s002.docx]

**S1 Table Primers Used in qRT-PCR**

| Marker | Forward primer (5’-3’) | Reverse primer (5’-3’) |
| --- | --- | --- |
| ABCG2 | AACCTGGTCTCAACGCCATC | GTCGCGGTGCTCCATTTATC |
| ΔNp63 | TCCATGGATGATCTGGCAAGT | GCCCTTCCAGATCGCATGT |
| N-cad | AGCCAACCTTAACTGAGGAGT | GGCAAGTTGATTGGAGGGATG |
| K14 | GACCATTGAGGACCTGAGGA | ATTGATGTCGGCTTCCACAC |
| K12 | CCAGGTGAGGTCAGCGTAGAA | CCTCCAGGTTGCTGATGAGC |
| Ki67 | CTTTGGGTGCGACTTGACG | GTCGACCCCGCTCCTTTT |
| GAPDH | CGACCACTTTGTCAAGCTCA | AGGGGTCTACATGGCAACTG |
