# Supplementary material for: Human adipose-derived stem cells support the growth of limbal stem/progenitor cells
Source: PLoS One. 2017 Oct 11;12(10):e0186238. doi: 10.1371/journal.pone.0186238 (PMC5636133; doi:10.1371/journal.pone.0186238)
Supplement: S2 Table — (DOCX) [file pone.0186238.s003.docx]

**S2 Table Primary Antibodies Used in Immunocytochemistry**

| Marker | Catalog No. | Company | Dilution |
| --- | --- | --- | --- |
| CD90 | ab133350 | Abcam | 1:100 |
| CD105 | ab114052 | Abcam | 1:200 |
| CD31 | ab28364 | Abcam | 1:30 |
| CD34 | ab6330 | Abcam | 1:20 |
| Adiponectin | ab113943 | Abcam | 1:100 |
| osteocalcin | ab13421 | Abcam | 1:100 |
| p63α | 4892S | Cell Signaling | 1:100 |
| K14 | K14 Ab(Clone LL002) | NeoMarkers/Fisher Scientific | 1:2 |
| K12 | Sc-25722 | Santa Cruz Biotechnology | 1:100 |
